# Chemokines: Orchestration of the Tumor Microenvironment and Control of Hepatocellular Carcinoma Progression

**DOI:** 10.1002/cam4.70789

**Published:** 2025-03-27

**Authors:** Jiezuan Yang, Haifeng Lu, Lanjuan Li

**Affiliations:** ^1^ The First Affiliated Hospital, Zhejiang University School of Medicine, State Key Laboratory for Diagnosis and Treatment of Infectious Diseases National Clinical Research Center for Infectious Diseases Hangzhou China

**Keywords:** chemokine, hepatocellular carcinoma, prognosis, receptor, tumor microenvironment

## Abstract

Chemokines, a family of chemotactic cytokines, play a central role in shaping the tumor microenvironment (TME) and in influencing the progression of hepatocellular carcinoma (HCC), a well‐known inflammation‐related cancer. This review addresses the intricate interplay between chemokines and HCC and highlights their multifaceted role. We discuss how altered expression of chemokines within the TME contributes to the development of HCC by orchestrating the recruitment of immune cells, ultimately leading to immunosuppression. In addition, we are investigating the contribution of chemokines to important features of HCC progression, including angiogenesis and epithelial‐mesenchymal transition (EMT). The potential of chemokines as serum biomarkers for HCC diagnosis and their potential as novel therapeutic targets are also explored. This comprehensive review emphasizes the importance of chemokines in the pathogenesis of HCC and their potential for a better understanding and treatment of this difficult disease.

## 1 Introduction

Chemokines are a class of low molecular weight proteins that have similar structures and functions and possess chemoattractive properties. They are the largest members of the cytokine family. They can be divided into four categories according to the different position of the two conserved N‐terminal cysteine residues: CC, CXC, C, and CX3C [1]. To date, more than 50 chemokines and about 20 chemokine receptors belonging to the G protein‐binding receptor family have been discovered. Many chemokines bind to different receptors and vice versa [1, 2]. In general, when chemokines bind to their receptors, they primarily play a role in the migration of white blood cells such as monocytes, eosinophils, and dendritic cells; some chemokines can also regulate the migration of endothelial cells and smooth muscle cells. Many human tumors are regulated by complex chemokine networks [3]. Under the action of specific chemokines, different subsets of immune cells invade the tumor microenvironment (TME) and regulate the immune response of the tumor [4]. Therefore, chemokines can directly or indirectly influence and shape the TME and thereby affect the biological behavior of tumors, including tumor growth, treatment, and patient prognosis.

Hepatocellular carcinoma is one of the most easily metastasizing cancers, associated with a high mortality rate of all cancers worldwide. It is the most common form of primary liver cancer and accounts for 90% of all liver cancer cases [5]. Epidemiological studies have shown that the prognosis of HCC patients is poor, with a median survival time of 11 months and a 1‐year survival rate of less than 50% for all cases [6]. In early‐stage HCC, the tumor can be effectively removed, and patient survival is relatively long. However, for late‐stage HCC, the survival time of patients is significantly shortened due to metastasis [7]. For advanced HCC patients, standard chemotherapy is ineffective, and the main treatment methods include not only liver resection and liver transplantation but also other treatment methods such as arterial chemoembolization (TACE), ethanol injection, radiofrequency or microwave ablation, immunotherapy, and selective in vivo radiation therapy (SIRT) [8]. In addition, traditional Chinese medicine (TCM) is sometimes effective in the treatment of liver cancer tumors, but the mechanism behind this is not yet clear [9].

In the tumor microenvironment (TME), chemokines can be expressed by various cells, including tumor cells, immune cells, and stromal cells. As chemotactic agents, chemokines play a crucial role in coordinating the infiltration of immune cells into the TME and in the spatiotemporal regulation of the tumor immune response [10]. In addition, chemokines can also directly affect non‐immune cells within the TME, such as tumor cells and vascular endothelial cells. Furthermore, chemokines exert both direct and indirect effects on tumor immunity and tumor growth, and thus have a significant impact on tumor development, treatment, and prognosis [11]. This review summarizes the current state of research on the effects of the major chemokine/receptor axes on the occurrence and prognosis of HCC. For ease of reading, the abbreviations, full names, and aliases of the chemokines are listed in Table 1, and the characteristic parameters and functions of the chemokines discussed in this review are listed in Table 2.

**TABLE 1:** Abbreviations, full names and aliases for the chemokines.

Abbreviation	Full name	Alias
CCL2	C‐C motif chemokine ligand 2	Monocyte Chemoattractant Protein‐1 (MCP‐1)
CCL4	C‐C motif chemokine ligand 4	Macrophage Inflammatory Protein‐1 beta (MIP‐1β)
CCL5	C‐C motif chemokine ligand 5	Regulated on Activation, Normal T cell Expressed and Secreted (RANTES)
CCL15	C‐C motif chemokine ligand 15	Hepatocellular carcinoma‐derived chemokine 4 (HCC‐4)
CCL20	C‐C motif chemokine ligand 20	Lymphotactin‐2 or macrophage inflammatory protein‐3 alpha (MIP‐3α)
CXCL1	C‐X‐C motif chemokine ligand 1	Melanoma growth stimulating activity alpha (GROα), GRO1 oncogene
CXCL2	C‐X‐C motif chemokine ligand 2	Macrophage Inflammatory Protein‐2 alpha (MIP‐2α)
CXCL3	C‐X‐C motif chemokine ligand 3	Melanoma growth stimulating activity gamma (GROγ), GRO3 oncogene, MIP‐2β
CXCL5	C‐X‐C motif chemokine ligand 5	Epitenoncyte‐activating peptide‐78 (ENA‐78)
CXCL8	C‐X‐C motif chemokine ligand 8	Interleukin‐8 (IL‐8)
CXCL9	C‐X‐C motif chemokine ligand 9	Monokine induced by gamma interferon (MIG) or Interferon‐gamma‐inducible protein 9 (IP‐9)
CXCL10	C‐X‐C motif chemokine ligand 10	Interferon‐gamma‐inducible protein 10 (IP‐10)
CXCL12	C‐X‐C motif chemokine ligand 12	Stromal Cell‐Derived Factor 1 (SDF‐1)
CXCL18	C‐X‐C motif chemokine ligand 18	Pulmonary and Activation‐Regulated Chemokine (PARC)
CX3CL1	C‐X3‐C motif chemokine ligand 1	Fractalkine

**TABLE 2:** Characteristic parameters and functions of chemokines in HCC.

Ck	Size (kDa)	Family category	Sources	Function in HCC	Ref.
CCL2	7.8	CC	Monocytes, Mφ, Endothelial cells	Promotes tumor growth and metastasis by inducing angiogenesis and recruiting immune cells. Inhibits apoptosis and induces cell proliferation	[12]
CCL4	7.8	CC	T cells, NK, Mφ	Contributes to tumor progression by affecting immune cell infiltration and angiogenesis	[13]
CCL15	7.8	CC	Monocytes, Mφ, Neutrophils	Involved in the recruitment of immune cells to the tumor site, potentially promoting tumor progression	[14]
CCL20	7.8	CC	T cells, Epithelial cells, Endothelial cells	Promote tumor growth and metastasis by influencing the tumor microenvironment and immune cell recruitment	[15]
CXCL1	7.8	CXC	PMNs, Endothelial cells	Contributes to tumor angiogenesis and metastasis by affecting endothelial cell and neutrophil functions	[16]
CXCL2	7.8	CXC	PMNs, Endothelial cells	Similar to CXCL1, promotes tumor angiogenesis and metastasis	[17]
CXCL10	7.8	CXC	Monocytes, Mφ, T cells	Pro‐tumor and anti‐tumor effects depending on the context; generally, it contributes to inflammation and immune cell recruitment	[18]
CXCL12	7.8	CXC	Endothelial cells, Stromal cells, Cancer cells	Promotes tumor growth, survival, and metastasis by influencing the tumor microenvironment and cell migration.	[19]
CX3CL1	31	CX3	Endothelial cells, Monocytes	Leukocyte recruitment and inflammation, potentially promoting tumor progression	[20]

*Note:* The role of chemokines often includes modulation of the tumor microenvironment, recruitment of immune cells, promotion of angiogenesis, and direct effects on cancer cell behavior. In addition, the functions of these chemokines in HCC can be complex and vary depending on the stage of the disease, the specific tumor microenvironment and the presence of other signaling molecules. Their role can range from promoting tumor development and metastasis to potentially inhibiting tumor growth, depending on the context and chemokine‐receptor interactions.

Abbreviations: HCC, hepatocellular carcinoma; Mφ, macrophage; NK, natural killer cell; PMNs, Polymorphonucleas.

## 2 Chemokines/Receptors Axes

### 2.1 CCL2/CCR2

C‐C motif chemokine ligand 2 (CCL2), also known as monocyte chemotactic protein‐1 (MCP‐1), is a major member of the chemokine CC subfamily. It was first isolated and purified from the culture supernatants of human glioma cells and human blood monocytes in 1989 [12]. Functionally, after binding to the receptor CCR2, CCL2 can activate signaling pathways such as the phosphatidylinositol 3‐kinase/protein kinase B (PI3K/AKT) pathway, the RAC‐GTPase pathway, the PKC (protein kinase C)‐dependent pathway, and the JAK/STAT (Janus kinase/signaler and activator of transcription) pathway, and thus exert various biological effects [21]. In addition, CCL2 promotes invasion and epithelial‐mesenchymal transformation (EMT) of HCC cell lines, which are associated with the activation of the Hedgehog (Hh) signaling pathway. Thus, the CCL2/CCR2 axis and the Hh signaling pathway play an important role in the progression of HCC, and CCL2 can also stimulate proangiogenic effects that further promote liver cancer progression [22]. In terms of diagnosis and treatment, Haag et al. suggested that CCL2 could serve as a potential marker for patient survival after selective internal radiation therapy (SIRT) [23]. In addition, the aqueous extract of aconite used in traditional Chinese medicine (TCM) inhibits the growth of HCC by CCL2‐dependent enhancement of natural killer cell infiltration [24].

### 2.2 CCL4, CCL5/CCR5

The common receptor of CCL4 and CCL5 is CCR5, which may play some role in liver cancer by recognizing and activating CCR5 [13]. Studies on the CCL5/CCR5 axis have shown that the expression of CCL5/CCR5 is significantly increased in HCC tissue compared to non‐tumorous liver tissue. The interaction between CCL5 and CCR5 can promote the proliferation and metastasis of cancer cells by enhancing protein kinase B (PKB, AKT) and EMT, while inhibition of the interaction between CCL5 and CCR5 can lead to apoptosis of HCC cells [8]. Cancer‐associated fibroblasts (CAFs) are one of the most common components of the HCC microenvironment and are closely related to HCC metastasis and invasion. Hu et al. [25] confirmed that CCL5 from CAFs can inhibit ubiquitination and degradation of hypoxia‐inducible factor 1 alpha (HIF1α) by binding to specific receptors, thereby maintaining HIF1α under normal oxygen conditions, upregulating the downstream gene zinc finger enhancer‐binding protein 1 (ZEB1), and inducing EMT, which ultimately promotes the lung metastatic ability of HCC cells.

Circular RNA studies revealed that circETFA in HCC can prolong the half‐life of CCL5 mRNA by recruiting eukaryotic translation initiation factor (EIF) 4A3 and acting as a sponge for hsa‐miR‐612 to inhibit the silencing effect of hsa‐miR‐612 on CCL5, thereby promoting HCC progression by increasing CCL5 expression [26]. In addition, has‐circ‐0003410 was shown to promote HCC progression by augmenting the proportion of M2/M1 macrophages via the miR‐139‐3p/CCL5 axis [27]. However, studies have also suggested that CCL4/CCL5/CCR5 may play some role in anti‐tumor therapy. Zhao et al. [28] reported that CCL4/CCL5 can interact with its receptor CCR5 and promote the recruitment of γδ‐T cells from the peripheral blood or surrounding tumor to the tumor area, thereby increasing the number of γδT cells in the tumor and improving the anti‐tumor immunity of γδ T cells and the prognosis of the patient.

### 2.3 CCL15/CCR1, CCR3

CCL15 is the most frequently expressed chemokine in human HCC. It not only promotes tumor invasion in an autocrine manner, but it can also specifically recruit monocytes with CCR1+ CD14+. Meanwhile, Liu et al. found that the CCL15/CCR1 axis forms a complex tumor‐promoting inflammatory microenvironment in human HCC and that blocking the CCL15/CCR1 axis in HCC may be an effective method for cancer treatment [14]. Wang et al. suggested that SPP1+ macrophages cooperate in promoting cancer stem cells via vitronectin and CCL15 signaling in liver cancer [29]. Furthermore, CCL15 expression has been shown to be associated with HBx in HCC patients, and CCL15 can be considered an indicator for the clinical treatment of HBV‐associated HCC [30]. In summary, the number of monocytes correlates positively with the expression of CCL15 and is an independent predictor of prognosis. In addition, Liu et al. show that MyD88 in myofibroblasts can promote hepatocarcinogenesis associated with non‐alcoholic fatty liver disease (NAFLD) by enhancing M2 polarization of macrophages [31].

### 2.4 CCL20/CCR6

In recent years, research has confirmed that the CCL20/CCR6 axis plays a critical role in promoting tumor growth in the tumor chemokine network [32]. Several lines of evidence suggest that CCL20/CCR6 can promote HCC cell proliferation, adhesion, migration, and invasion [15]. It has been shown that high expression of CCL20 in HCC is associated with poor patient prognosis. High expression of CCL20 not only promotes the appearance of EMT in HCC cells, but also activates STAT3 to induce Th9 helper T cells with tumor‐promoting effects targeting the HCC tumor microenvironment [33]. In addition, CCL20 expression is associated with HCC recurrence and patient survival, which may be expressed through the PI3K/AKT and Wnt/β‐catenin signaling pathways, which induce EMT and promote HCC cell proliferation and migration [34]. One study found that CD4^+^ and CD8^+^ T lymphocytes expressing CCR6 were significantly reduced in the peripheral blood of HCC patients, while CCR6 was highly expressed in tumor‐infiltrating lymphocytes and adjacent non‐tumor‐infiltrating lymphocytes of the liver, suggesting that CCR6 plays an important role in the recruitment of lymphocytes from peripheral blood to HCC [35].

Similarly, Th17 cells expressing CCR6 were found to significantly increase in HCC tumor tissue, and the density of CCR6+ Th17 cells was associated with overall survival and disease‐free survival of HCC patients. The accumulation of CCR6+ Th17 cells may also promote the progression of HCC by inducing angiogenesis [36]. In addition, a study by Wang et al. [37] demonstrated the function of the HOXD3‐CREBBP/Med15‐CCL20‐CCR6 axis in regulating invasion and migration in HCC. Tumor‐associated macrophages (TAMs) are considered anti‐tumor suppressors, and in HCC, TAMs can regulate kinase (ERK)/NF through extracellular signaling (the κ‐B pathway), which increases the expression of CCL20 and subsequently induces the accumulation of CCR6+ Tregs, which may lead to resistance to programmed death ligand 1 (PD‐L1) immunotherapy [38].

### 2.5 CXCL1, CXCL3, CXCL5, CXCL8/CXCR2

Research has confirmed that high expression of CXCL1 can predict recurrence in HCC patients and that CXCL1 can promote the development of HCC by increasing mitochondrial metabolism and inducing EMT [16]. CXCL1 signal transduction and CXCR2 binding may stimulate HCC cell recovery from apoptosis or influence the metastatic potential of HCC cells by promoting migration [39]. The combination of expression levels is a strong predictor of poor prognosis in HCC patients. Studies on CXCL3 have confirmed that serum CXCL3 levels are higher in HCC patients than in healthy individuals. High CXCL3 levels are significantly associated with poor prognosis, vascular invasion, and tumor capsule formation in HCC patients [40]. However, Li et al. show that the CRNDE‐NF‐κB‐CXCL3 axis plays a critical role in controlling the immunosuppressive niche that promotes HCC progression through the recruitment of G‐MDSCs [41].

In addition, the CXCL5/CXCR2 axis has been shown to activate the PI3K/AKT/GSK‐3β (glycogen synthase kinase‐3β)/Snail (human snail homolog (Drosophila)–like 1 protein) signaling pathway, which is involved in the EMT of HCC and significantly increases the proliferation, migration, and invasiveness of HCC cells [42]. In addition, Ren et al. identified the Sox9/CXCL5 axis as an endogenous factor in the control of HCC cell growth and invasion [43]. In terms of diagnosis, Laschtowitz et al. found that CXCL5 can serve as a diagnostic biomarker for early detection of HCC in cirrhotic patients as well as for tumor progression, regardless of the underlying liver disease [44].

The chemokine CXCL8, also known as interleukin‐8 (IL‐8), is a cytokine secreted by macrophages and epithelial cells. Interleukin‐8 binds to the chemokine receptors interleukin‐8 receptor‐alpha (IL8RA, also known as CXCR1) and interleukin‐8 receptor‐beta (IL8RB, also known as CXCR2) and has a chemotactic effect on neutrophils to regulate their inflammatory response. The results of Ma et al. [45] show that IFN‐α causes immunosuppression and cancer stem cell differentiation in hepatocellular carcinoma by upregulating CXCL8 secretion. Zhao et al. [46] also show that low‐dose metformin can inhibit HCC metastasis by suppressing IL‐8 expression. CXCL8 was also found to be significantly upregulated in HCC, promoting tumor progression and metastasis through activation of the AKT/mammalian rapamycin target protein (mTOR)/STAT3 pathway [47].

### 2.6 CXCL2/CXCR7

CXCL2 is a class of chemokines that primarily has a chemotactic effect against neutrophils. In combination with its receptor CXCR7, it can chemotactically attract neutrophils and promote the growth and metastasis of some tumors [17]. CXCR7 is highly expressed in tumor endothelial cells and promotes HCC migration and invasion by influencing the phosphorylation of the STAT3 pathway [48]. Remarkably, CXCL2 also showed activity against the atypical chemokine receptor 2 (ACKR2), although this was weak compared to CXCL10 or the activity against its classical receptor. CXCL2 has no scavenger receptor and is an important inflammatory chemokine and a potent neutrophil chemoattractant. Interestingly, it was recently reported that ACKR2‐deficient mice exhibit increased neutrophil infiltration in various tissues and higher neutrophil antimetastatic activity than normal mice [49]. It remains to be investigated whether the enhancement of these neutrophil processes is due to the suppression of CXCL2 regulation by ACKR2.

### 2.7 CXCL9/CXCR3

The chemokine CXCL9 and its receptor CXCR3 are increasingly recognized as important players in the pathogenesis and progression of HCC [50]. CXCL9, which belongs to the CXC chemokine family, mediates its effect mainly via CXCR3, which is found primarily on activated T cells, natural killer cells, and cancer cells. Recent studies have emphasized the dual role of the CXCL9/CXCR3 axis in HCC. On the one hand, CXCL9/CXCR3 promotes anti‐tumor immunity by attracting CXCR3+ cytotoxic lymphocytes to the tumor microenvironment, which can inhibit tumor growth [51]. For example, one study showed that high intratumoral CXCL9 levels correlate with increased T‐cell infiltration and better prognosis in HCC patients. On the other hand, some studies suggest that CXCL9/CXCR3 may contribute to tumor progression and metastasis [52]. Thus, the role of CXCL9/CXCR3 in HCC is complex, suggesting that its therapeutic potential may depend on the specific tumor context or setting. Targeting the CXCL9/CXCR3 axis may be a novel approach to modulate the immune situation in HCC, although more detailed mechanistic studies and clinical trials are still needed to fully understand its therapeutic impact and efficacy.

### 2.8 CXCL10/CXCR3

CXCL10 has a dual effect on tumor development, depending on the splice variant of the corresponding CXCR3 receptor. CXCR3‐B has growth inhibitory properties, while CXCR3‐A promotes cell proliferation [18]. CXCL10 binds to CXCR3‐B receptors on the surface of tumor cells and thus directly inhibits proliferation and shortens the survival time of tumor cells; binding to the CXCR3‐A receptor attenuates FAS (CD95)‐mediated cell apoptosis, tumor cells secrete CXCL10, and chemotactic CXCR3+ CD4/CD8/Treg cells invade the tumor tissue [53]. In addition, our group reported that CXCL10 and MIP‐3a (CCL20) are more sensitive than AFP as diagnostic biomarkers for HCC [54]. In addition, IP‐10 and CXCR3 are associated with anti‐tumor immunity in HCC patients and represent a potential target for the treatment of HCC [55]. Enhanced CXCL10/CXCR3 signaling after acute liver transplantation directly leads to the mobilization and recruitment of Treg cells, thereby promoting the growth and recurrence of HCC after transplantation [56]. Therefore, certain tumor cells with a high expression of CXCL10 and CXCR3 have stronger metastatic and invasive capabilities.

### 2.9 CXCL12/CXCR4, CXCR7

The CXCL12/CXCR4 axis is considered an important factor in the regulation of angiogenesis, which is crucial for the growth and progression of HCC. Using immunohistochemical techniques, Li et al. [57] used online databases for analysis and investigated cellular communication between TAMs and T cells, revealing potential signaling pathways such as the CXCL10/CXCL11‐CXCR3 and CXCL12‐CXCR4 networks in HCC patients. Lu et al. [19] suggested that the expression level of its receptor should be considered when evaluating the prognostic significance of CXCL12 in HCC, and CXCL12 could potentially serve as a promising prognostic marker for HCC. In addition, abnormally high expression of CXCR4 was detected in the capillaries of 50% of HCC cases 6. The results suggest that the CXCL12/CXCR4 axis may play an important role in the progression of HCC by promoting neovascularization. Moreover, the strong expression of CXCR4 also correlates significantly with the clinical and pathological features of HCC [58].

In addition, other studies have also confirmed that the increased expression of CXCR4 in HCC tissues is related to tumor size, metastasis, and patient survival [59]. However, some studies suggest that there is no significant association between CXCR4 and the occurrence and development of HCC, while the CXCL12 gene polymorphism is associated with the occurrence and development of HCC [60]. In addition, Morita et al. [61] pointed out that combined CXCR4/PD1 blockade can reprogram intra‐tumoral cDC1s and has the potential to enhance the anti‐tumor immune response against HCC. In addition, CXCL12 from hepatic stellate cells can also increase their migratory activity in vitro by inducing EMT in HCC cells, and Flavokawain A could suppress this process [62]. Currently, the CXCL12/CXCR4 axis is thought to involve several tumor‐related signaling pathways in the HCC process, such as nuclear factor (NF)‐κ B, PI3K/protein kinase B (AKT), and c‐Jun amino‐terminal kinase (JNK) [63].

### 2.10 CX3CL1/CX3CR1

CX3CL1 is the only member of the CX3C chemokine subfamily and can occur in vivo in two forms: membrane‐bound and secretory. CX3CR1 is the sole receptor for CX3CL1, and the CX3CL1/CX3CR1 axis has been shown to regulate functions such as inflammatory cell chemotaxis and tumor cell adhesion. This system can have both tumor‐inhibiting and tumor‐promoting effects [20]. Heo et al. [64] found that the extracellular vesicles of breast cancer cells induce TNF‐α expression in the liver, leading to upregulation of CX3CL1. Finally, CX3CL1 plasma levels were significantly associated with the development of liver metastases in 155 breast cancer patients. HCC research has found that upregulation of CX3CL1/CX3CR1 expression can be detected in spinal metastases and that bone marrow endothelial cells can promote the migration and invasion of HCC cells into the spine through the secretion of soluble CX3CL1. In addition, CX3CL1 secreted by bone marrow endothelial cells can also promote the growth of metastatic HCC cells in the spine [65].

In contrast to the above‐mentioned research findings on the promotion of HCC by the CX3CL1/CX3CR1 axis, Chen et al. [66] found that low CX3CL1 levels and high miR‐561‐5p levels in HCC tissues were associated with lung metastases and poor prognosis. Mechanism studies have shown that CX3CL1 exerts its anti‐HCC effect by signaling CX3CR1+ natural killer (NK) cell chemotaxis and cytotoxicity via transcription activation factor 3 (STAT). miR‐561‐5p can downregulate the expression of CX3CL1 mRNA, leading to decreased infiltration and regulatory function of CX3CR1+ NK cells, thereby promoting HCC growth and lung metastasis. Similarly, Miao et al. [67] confirmed that the CX3CR1/PI3K signaling pathway plays a crucial role in CXCL3‐induced platelet migration. HCC cell‐derived CX3CL1 facilitates platelet infiltration into the tumor and subsequently triggers apoptosis of HCC cells.

## 3 Summary and Outlook

### 3.1 Specific Chemokines Influence Different Immune Cell Types

In general, chemokines such as CCL2, CCL4, and CXCL10 can recruit cytotoxic T lymphocytes (CTLs) to the tumor and thus promote the anti‐tumor response. CXCL13 is known to attract B cells to lymphoid tissue, and its role in HCC would require further investigation [68]. Chemokines such as CXCL10 and CX3CL1 can attract NK cells that can directly kill tumor cells. However, CCL22 produced by tumor cells or other cells in the TME can attract Tregs, which contribute to the suppression of anti‐tumor immunity [69]. Chemokines such as CCL2, CCL5, and CXCL12 are known to recruit myeloid‐derived suppressor cells (MDSCs) to the tumor microenvironment, where they suppress T‐cell responses and promote tumor growth [70]. Furthermore, the investigation of the specific chemokine receptor axes that play a role in HCC is of crucial importance for understanding the complex interplay between the tumor and the immune system. This knowledge can then be used to develop new therapeutic strategies targeting these signaling pathways to improve anti‐tumor immunity.

### 3.2 Chemokines Affect TME Components

Chemokines orchestrate a delicate dance between tumor cells and surrounding cells such as fibroblasts, endothelial cells, and macrophages, and ultimately influence the progression of HCC. CCL2 (MCP‐1) acts like a siren and lures hepatic stellate cells (HSC), the precursors of cancer‐associated fibroblasts (CAF), into the tumor [71]. Once there, CCL2 stimulates these HSCs to transform into activated CAFs, the master builders of the TME. Once the CAFs are established, CXCL12 increases their production of extracellular matrix (ECM) components such as collagen. This process, known as fibrosis, leads to the stiffening of the tumor and creates a physical barrier that impedes drug delivery and immune cell infiltration. In addition, CXCL8 (IL‐8) acts as a powerful signaling agent that attracts endothelial cells to the tumor and stimulates them to form new blood vessels. This process, known as angiogenesis, supplies the tumor with important nutrients and oxygen for rapid growth [72]. In addition, CCL2 plays a dual role in the TME. First, it attracts macrophages to fight the tumor, and second, it can induce them to adopt a tumor‐friendly phenotype, the M2 macrophages. CXCL12 can further polarize macrophages towards the M2 type, suppressing the anti‐tumor immune response and promoting tumor growth.

### 3.3 Potential Side Effects of Targeting Specific Chemokines

Chemokines are not only attractants for immune cells; they also control crucial processes throughout the body. They control cell migration during development, regulate stem cell activity during tissue repair, and influence organ homeostasis [73]. Disruption of these functions could lead to unintended consequences such as wound healing disorders, developmental abnormalities, or even increased susceptibility to infections [74]. To mitigate these risks, several considerations are critical when selecting chemokine targets: (1) Specificity: select chemokines with limited expression patterns and specific roles in the disease process, and exclude chemokines with widespread functions. (2) Redundancy: the chemokine system often has built‐in redundancy. Blocking a single chemokine can trigger compensatory mechanisms and limit efficacy. Targeted combinations can be more effective, but they also increase the risk of side effects. (3) Drug delivery: localized delivery methods, such as topical application or targeted injections, can help limit the effect of the drug to the diseased area and minimize systemic exposure. (4) Alternative strategies: rather than blocking the chemokine itself, targeting downstream signaling pathways that are activated by the chemokine‐receptor interaction may provide greater specificity.

### 3.4 Future Research Directions on Chemokines in HCC

Future research on chemokines in HCC could focus on the use of advanced multi‐omics approaches to gain deeper insights into their role within the tumor microenvironment (TME) [75]. For example, single‐cell RNA sequencing (scRNA‐seq) can be used to uncover the cell‐specific expression patterns of chemokines and their receptors and provide a detailed map of intercellular signaling networks [76]. Second, metabolomics could elucidate the interplay between chemokine signaling pathways and metabolic reprogramming in HCC and shed light on how chemokines influence tumor metabolism and immune response. The integration of scRNA‐seq, metabolomics, and other omics data (e.g., proteomics, epigenomics) may help to identify novel chemokine‐related therapeutic targets and biomarkers by revealing functional interactions between chemokines, immune cells, and stromal components in the TME. In addition, the study on the effect of traditional Chinese medicine (TCM) on chemokines in the treatment of HCC will also help to clarify the mechanism of TCM in the treatment of tumors.

### 3.5 Chemokines and Metabolism

Chemokines have been shown to influence key metabolic processes such as glucose uptake, glycolysis, and fatty acid oxidation, which are critical for tumor growth and immune cell function in the TME [77]. Conversely, metabolic reprogramming can also influence the expression and function of chemokines, creating a reciprocal feedback loop. For example, hypoxia‐induced metabolic changes can upregulate certain chemokines, promoting tumor growth and immune evasion [78]. However, incorporating this bidirectional relationship would lead to a more comprehensive understanding of the complexity of the TME. Further discussion of how chemokine‐induced metabolic changes affect immune cells such as T cells or macrophages, and how metabolic changes in turn shape chemokine gradients, could greatly enrich the study's insights into tumor biology and potential therapeutic strategies.

### 3.6 Microbiome Regulating Chemokines

Emerging evidence suggests that the human microbiome plays a critical role in modulating the immune system, including the regulation of chemokines [79]. Chemokines, signaling proteins that control the transport of immune cells, are crucial for maintaining immune homeostasis and responding to pathogens. The microbiome can influence the production and function of chemokines through several mechanisms: (1) Metabolite production: metabolites derived from the microbiome, such as short‐chain fatty acids, can suppress or enhance the expression of chemokines in immune cells. (2) Signaling effect on epithelial cells: the signaling effect on epithelial cells mediated by the microbiome can lead to the production of chemokines that attract immune cells to the site of action. (3) Modulation of immune cells: the microbiome can influence the function and phenotype of immune cells and alter their chemokine production profiles. Conversely, dysregulation of this microbiome–chemokine axis can contribute to various inflammatory and autoimmune diseases, highlighting the potential for microbiome‐targeted therapies [80].

### 3.7 Synergistic or Antagonistic Effects of Different Chemokine Axes

Chemokines and their receptors, acting as axes such as CXCL12/CXCR4, play a crucial role in shaping the tumor microenvironment (TME) and influence the progression of hepatocellular carcinoma (HCC) [81]. These axes may exhibit synergistic or antagonistic effects and modulate tumor growth, immune infiltration, and metastasis. For example, the CXCL12/CXCR4 axis promotes tumor proliferation, angiogenesis, and immune evasion by recruiting immunosuppressive cells such as regulatory T cells (Tregs) and myeloid‐derived suppressor cells (MDSCs). Synergistically, the CCL2/CCR2 axis reinforces this immunosuppressive milieu by attracting macrophages with tumor‐promoting phenotypes [82]. Conversely, some axes may act antagonistically. For example, CXCL9/CXCR3, which is known to recruit cytotoxic T cells, may counteract the tumor‐promoting effects of CXCL12/CXCR4 by enhancing anti‐tumor immunity [83]. The interplay of these axes creates a dynamic TME in which the balance between pro‐ and anti‐tumor signaling determines HCC progression. Simultaneously targeting multiple chemokine axes may provide therapeutic strategies to disrupt this balance and suppress the development of HCC.

### 3.8 Chemokine Targeting and Resistance

The targeted use of chemokines as a therapeutic strategy can unintentionally lead to resistance via various mechanisms [84]. One common pathway is the compensatory activation of alternative chemokine signaling pathways. When a particular chemokine or its receptor is inhibited, other chemokine‐receptor pairs with overlapping functions may be upregulated and maintain tumor‐promoting processes such as angiogenesis, immune response, or metastasis. For example, blocking CXCL12‐CXCR4 signaling may lead to compensatory activation of the CXCL8‐CXCR2 axis that maintains tumor progression [85]. In addition, tumors may develop alternative strategies to evade the immune system. For example, targeting chemokines involved in T cell recruitment, such as CXCL9 or CXCL10, could lead to upregulation of immunosuppressive signaling pathways, such as increased infiltration of regulatory T cells (Tregs) or myeloid‐derived suppressor cells (MDSCs) [86]. These mechanisms highlight the complexity of chemokine networks and the need for combination therapies to overcome resistance and effectively disrupt tumor‐promoting pathways.

**FIGURE 1:**
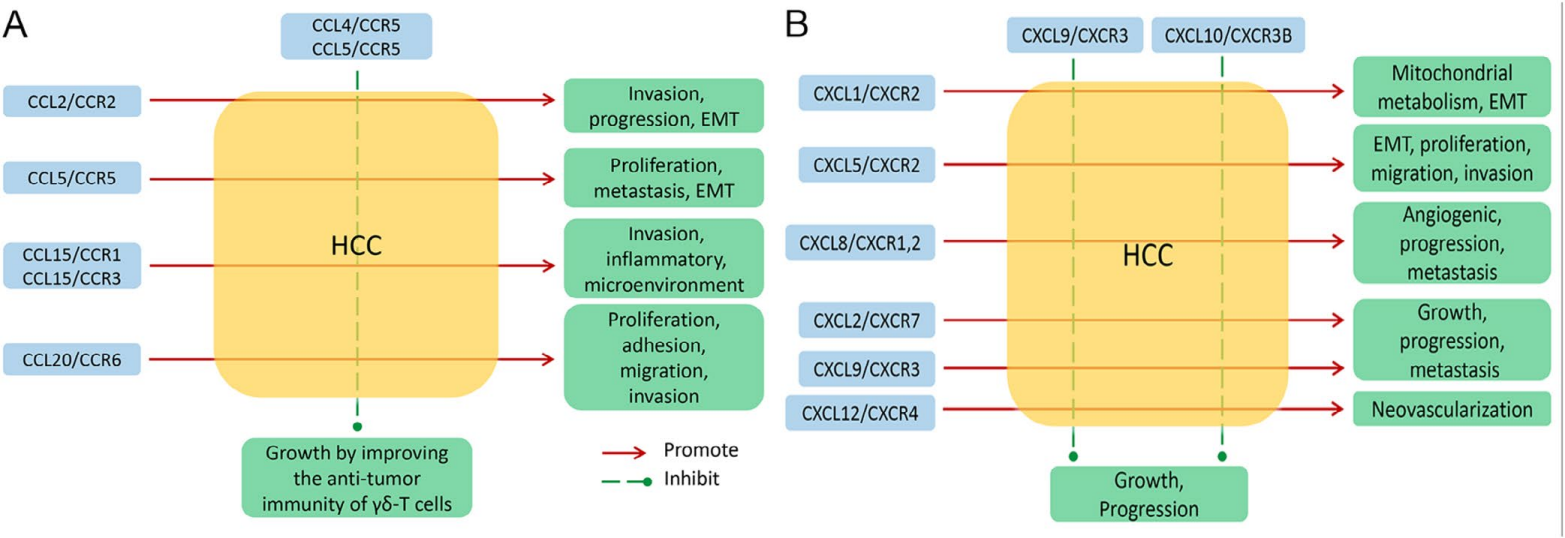
Chemokines and receptors of the CC and CXC subfamilies regulating the different behaviors of HCC. The CC (A) and CXC (B) subfamily of chemokines and their receptors are presented respectively. (A) shows that, except for the inhibitory effect of CCL4/CCR5 and CCL5/CCR5 on HCC growth, most other CC subfamily chemokines and their receptors promote HCC invasion, EMT, proliferation, or metastasis. Similarly, (B) shows that, except for the inhibitory effect of CXCL9/CXCR3 and CXCL10/CXCR3B on HCC growth, most other CXCL subfamily chemokines and their receptors promote HCC migration, EMT, metastasis, or neovascularization. In addition, CXCL9/CXCR3 inhibits tumor growth by attracting CXCR3+ cytotoxic lymphocytes, but may also contribute to tumor progression and metastasis. Furthermore, the role of chemokines in HCC can be complex and vary depending on the stage of disease, the specific tumor microenvironment, and the presence of other signaling molecules. EMT, epithelial‐mesenchymal transformation.

In summary, the complex network of chemokines and their receptors plays an important role in the development and metastasis of HCC; a schematic diagram of their interaction is shown in Figure 1. The changes in the expression of chemokines at different stages of HCC are summarized in Table 3. Although considerable progress has been made in understanding the HCC microenvironment and many studies have been conducted on various chemokines [34]. Although the role of specific chemokine‐receptor axes is particularly important in liver cancer; the exact mechanism needs further research, and some contradictory conclusions cannot be explained [87]. Although the role of chemokines in the development of liver cancer is not yet fully understood, they are likely to bring more benefits to liver cancer patients by regulating local chemokines in liver cancer and combining them with immunotherapies [88]. However, there are other chemokines that play an important role in the development of HCC, such as CCL16, which promotes the progression of HCC by recruiting monocytes and macrophages [89].

**TABLE 3:** Changes in the expression of chemokine/receptor at different stages of HCC.

Chemokine/receptor	Early HCC	Intermediate HCC	Advanced HCC
CCL2/CCR2	↑	↑	↑
CCL4/CCL5/CCR5	↑	↑	↑
CCL15/CCR1	↑	↑	↓
CCL20/CCR6	↑	↑	↓
CXCL1/CXCL3/CXCL5/CXCL8/CXCR2	↑	↑	↑
CXCL2/CXCR2	↑	↑	↓
CXCL9/CXCR3	↑	↑	↓
CXCL10/CXCR3	↑	↑	↓
CXCL12/CXCR4	↑	↑	↓
CX3CL1/CX3CR1	↑	↑	↓

*Note:* The expression of chemokines can vary depending on the specific population, stage and other factors.

Abbreviations: ↑, increased expression; ↓, decreased expression.

## Author Contributions

Jiezuan Yang and Haifeng Lu wrote the original draft and revised the manuscript. Lanjuan Li coordinated the study and the technical aspects of the work.

## Conflicts of Interest

The authors declare no conflicts of interest.

## Data Availability

Data sharing is not applicable to this article as no new data were created or analyzed in this study.
